# Organization of the Tennessee Dental Association

**Published:** 1867-11

**Authors:** 


### ORGANIZATION OF TENNESSEE DENTAL ASSOCIATION.

Pursuant to a call made at a preliminary meeting of the Dentists, of Memphis, on the 20th day of June, the following members of the profession convened at Nashville on the 26th of July, and organized the Tennessee Dental Association.

Present—Drs. W. H. Morgan, Nashville; G. W. Acree, Wm. T. Arrington, J. B. Wasson, Memphis; J. A. Arrington, Jackson; H. M. Acree, Clarksville; R. Russell, J. C. Ross, S. J. Cobb, W. P. Wilson, Nashville; Alex. Hartman, Murfreesboro; M. McCarty, Pulaski; T. E. Beech, Franklin; W. R. Johnston, Columbia.

At 10 A. M., with Dr. Morgan as Chairman, *pro tem.* and Dr.

Ross, Secretary, the house was called to order, and the following committee appointed to draft Constitution and By-laws for the Association: Drs. Wm. T. Arrington, Chairman, S. J. Cobb, W. R. Johnston.

A nominating committee was also appointed: Drs. J. B. Wasson, Chairman, Alex. Hartman, R. Russell.

While the committees were in session Drs. G. W. Acree, and W. H. Morgan addressed the meeting upon the subject of *Dental Education, State and Local Organizations*, and the importance of prompt and immediate action towards the general advancement of the Dental profession.

Committee on Constitution and By-laws presented a paper which was received and committee discharged. On motion the paper was taken up by sections, discussed and voted upon, and then voted and approved as a whole and adopted as the Constitution and By-laws under which to organize.

The Committee on Nomination made the following report:

Dr. W. H. Morgan, for President.

Dr. J. B. Wasson, First Vice President.

Dr. J. C. Ross, Second Vice President.

Dr. Wm. T. Arrington, Recording Secretary.

Dr. R. Russell, Corresponding Secretary.

Dr. Alex. Hartman, Treasurer.

Drs. G. W. Acree, J. A. Arrington, W. R. Johnston, Executive Committee.

The meeting adjourned for dinner and convened at 2½ P. M. An election was held and those nominated duly elected by ballot to serve as officers in the Association for the term of one year, and after being properly installed, the President made a few appropriate remarks, and then declared the Association organized and ready for business.

On motion of Dr. W. T. Arrington it was resolved that the Code of Ethics of the American Dental Association, be approved and adopted by this Association.

Adjourned to meet at 9 A. M. Saturday, 27th.

SATURDAY, July 27.

Society met. Minutes read and approved.

On motion it was resolved that the Semi-annual meeting be held at Jackson, from the 20th to the 24th of December next, and the Annual meeting to be held at Memphis, on Wednesday preceding the last Tuesday in July, 1868.

Drs. Morgan, Acree and Beech were duly elected delegates to the American Dental Association at Cincinnati.

Many interesting subjects were discussed, and the liveliest interest manifested by all present.

On motion it was resolved that the Secretary be requested to furnish the *Dental Cosmos*, the *American Journal of Dental Science*, and the DENTAL REGISTER, with a synopsis of the proceedings.

Adjourned at 12 M., to meet at Jackson on Friday, the 20th of December.

WM. T. ARRINGTON, *Rec. Sec'y.*

---

CINCINNATI, Nov. 7, 1867.

AT a meeting of the "share-holders," of the Association, to contest the claims of the "Goodyear Dental Vulcanite Company," convened in the lecture room of the Ohio Dental College, Dr. James Taylor was called to the Chair, Dr. W. P. Horton appointed Secretary.

Dr. J. Taft, in behalf of the Committee, made a brief statement, and at the close of his remarks Col. S. S. Fisher, Attorney for the Dentists, made a verbal report, after which, remarks were made by most present upon the general subject.

On motion of Dr. Cushing, the Chair appointed a committee of three, consisting of Drs. Geo. H. Cushing, J. Taft, and A. A. Blount, to report a resolution expressive of the sense of this meeting. The Committee, after consultation reported the following;

*Resolved*, That we approve the course pursued by the Executive Committee, and Attorney, who have been acting for the Dental profession of the West, in contesting the claims of the "Goodyear Dental Vulcanite Company," against the profession; and that we request them to continue the defense to the ultimatum, believing that notwithstanding the decision of Judge Nelson, in New York, the importance of the subject demands a full and final investigation by the highest tribunal in the Country.

On motion the report was accepted and adopted.

On motion adjourned *sine die*.

W. P. HORTON, Sec'y. JAMES TAYLOR, President.

THE following resolution was adopted at a meeting of the Chicago Dentists, held Monday evening, Nov. 11, 1867:

*Resolved*, That the Dental profession of Chicago approve the action of the Executive Committee of the Ohio State Dental Association, in defending the suits pending between the Goodyear Dental Vulcanite Company, and different members of the profession. And that we request them to continue their defense until a final decision of the Supreme Court of the United States, at Washington has been obtained.

